# LipidWrapper: An Algorithm for Generating Large-Scale Membrane Models of Arbitrary Geometry

**DOI:** 10.1371/journal.pcbi.1003720

**Published:** 2014-07-17

**Authors:** Jacob D. Durrant, Rommie E. Amaro

**Affiliations:** Department of Chemistry & Biochemistry, University of California San Diego, La Jolla, California, United States of America; Carnegie Mellon University, United States of America

## Abstract

As ever larger and more complex biological systems are modeled *in silico*, approximating physiological lipid bilayers with simple planar models becomes increasingly unrealistic. In order to build accurate large-scale models of subcellular environments, models of lipid membranes with carefully considered, biologically relevant curvature will be essential. In the current work, we present a multi-scale utility called LipidWrapper capable of creating curved membrane models with geometries derived from various sources, both experimental and theoretical. To demonstrate its utility, we use LipidWrapper to examine an important mechanism of influenza virulence. A copy of the program can be downloaded free of charge under the terms of the open-source FreeBSD License from http://nbcr.ucsd.edu/lipidwrapper. LipidWrapper has been tested on all major computer operating systems.

This is a *PLOS Computational Biology* Software Article

## Introduction

In recent years, an exponential increase in computer power has permitted computational biologists to generate and study increasingly larger and more complex biological systems *in silico*. Early efforts involved only small proteins; in contrast, multi-protein complexes as large as the entire ribosome have been recently modeled and studied [1], and virtual representations of entire viral particles are now becoming feasible as well [2], [3]. Technological advances in microscopy and other experimental techniques, which provide data of increasingly high quality [4], [5], inform model complexity [3], [6], [7], [8].

Many important biological processes occur on the surfaces of lipid bilayers. For example, roughly 30% of all FDA-approved drugs target G protein-coupled receptors [9], large proteins that span biological membranes and are responsible for transmitting a myriad of signals from the extracellular to the intracellular environment. Aside from mediating cell signaling, bilayers also provide the foundation for cellular structure and compartmentalization.

In the past, lipid bilayers have often been modeled as planar structures (see, for example, refs. [10], [11], and [12]). As the systems being modeled were small (e.g., with bilayers whose cross-sectional areas were less than 40,000 Å^2^), this approximation was acceptable. However, as models of increasing size and complexity are generated, the planar-bilayer model has become in many instances unrealistic. *In vivo*, membrane-bound proteins are embedded in curved bilayers that encompass entire cells or organelles. In some instances, lipid curvature itself is critical to biological function (e.g., viral budding and membrane scission [13], [14]). When modeling large, multi-protein systems that include interactions with a lipid bilayer, bilayer curvature must be carefully considered [15], [16], [17].

At present, atomistic and coarse-grained models of expansive subcellular structures such as cellular membranes cannot be obtained directly from experiment due to the “methodological gap” that exists between atomistic and lower-resolution imaging techniques [3], [7], [8]. For example, X-ray crystallography [18] and NMR [19] are capable of producing atomic-resolution models but are subject to restrictive size limitations. The average resolution of the X-ray structures deposited in the Protein Data Bank (PDB) [20] as of March 2014 was 2.2 Å; the best structure had a resolution of 0.48 Å [21]. However, with some notable exceptions (see, for example, refs. [22], [23]), structures deposited in the PDB represent complexes with at most only a few protein components. Ninety-five percent of the deposited X-ray crystallographic and solution-NMR structures had masses less than 237,000 and 22,500 Daltons, respectively.

In contrast, extensions of traditional transmission electron microscopy like electron and cryo-electron tomography are capable of producing three-dimensional models of larger multi-component systems. In some cases, when multiple geometrically similar particles are simultaneously imaged or when the same particle is imaged multiple times in rapid succession, spatial [24], [25] and/or temporal [26] averaging can achieve resolutions of 3 to 4 Å [27], [28]. However, in general electron tomography lacks the resolution of X-ray crystallography. The average resolution of the models deposited in the EMDataBank [29] as of March 2014 was 19.5 Å, though the best structure had an impressive resolution of 3.1 Å [30].

No current experimental technique can combine the advantages of these two regimes to produce atomistic or coarse-grained models of whole large-scale, multi-component subcellular environments. Rather, building these types of models requires researchers to assemble multiple crystallographic and/or NMR models *in silico* according to the larger-scale configurations dictated by electron tomography [3], [6], [7], [8].

To facilitate the incorporation of physiologically relevant large-scale lipid-bilayer models into these expansive multi-component representations, we recently created an easy-to-use computer program called LipidWrapper. LipidWrapper is unique among existing bilayer-modeling programs such as CHARMM-GUI's Membrane Builder [31], Packmol [32], and autoPack/cellPack [33], [34]. Rather than assembling models lipid by lipid, LipidWrapper extracts membrane patches from pre-equilibrated planar bilayer models and so produces curved models that, on the whole, are more physiological. Additionally, LipidWrapper is capable of generating models of arbitrary geometry and size (up to 1.3 cubic microns), requires no knowledge of a specialized scripting language, and has a helpful graphical user interface that improves usability.

We are hopeful that LipidWrapper will be a useful tool for the computational-biology community. The program, which requires python, *numpy*, and *scipy* [35], [36], [37], [38], [39], has been tested on all major operating systems (Table 1). A copy can be downloaded free of charge from http://nbcr.ucsd.edu/lipidwrapper.

**Table 1:** Operating-system compatibility.

Operating System	Python Version	*Numpy* Version	*Scipy* Version
Scientific Linux 6.2	2.6.6	1.6.2	0.11.0
OS X 10.8.3	2.7.2	1.6.1	0.12.0.dev-bd22c4b
Windows XP Professional	2.7.3	1.7.0rc1	0.11.0
Windows XP Professional	2.6	1.6.1rc1	0.9.0
Windows 7 Home Premium	2.7.6	1.8.0	0.13.3

LipidWrapper has been successfully tested on several different operating systems with various versions of python, *numpy*, and *scipy*.

## Design and Implementation

### Defining and Tessellating Mesh Points

**Figure 1:**
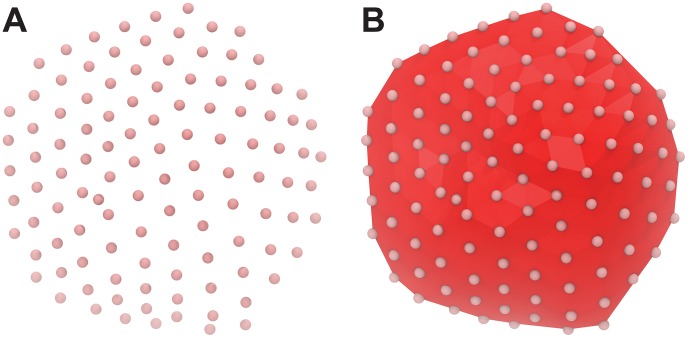
Tessellating the surface. Prior to generating a curved bilayer model, LipidWrapper must first characterize the requested geometry. A) Initially, simple mesh points define the bilayer surface. B) If triangulation is not provided (i.e., a collada DAE file is not specified), LipidWrapper subsequently tessellates these points so that the entire surface is covered in small triangles.

The LipidWrapper algorithm begins by considering mesh points that define a bilayer surface (Figure 1A). These mesh points can be generated in four ways: the user can provide a PDB file with atomic coordinates that define the points; the points can be defined by a gray-scale image, where black is topologically “low” and white is topologically “high”; the user can provide a function that defines a single *z* value for any (*x*, *y*) coordinate; and the user can provide a collada DAE file with both the mesh points and surface polygons (triangles) specified [40].

For the first three of these options, a tessellated/triangulated surface must be generated from the mesh points. All mesh points are projected onto the *x*–*y* plane, and the resulting set of coplanar points is subjected to a 2D Delaunay triangulation [41]. This triangulation is then projected back onto the 3D mesh (Figure 1B). As the triangulation requires an intermediate 2D projection, bilayer models that cover concave or enclosed surfaces are not permitted.

For complex shapes, including concave and enclosed surfaces, the user can provide a collada DAE file [40] with the surface tessellation/triangulation already specified. LipidWrapper has been specifically tested with collada files exported from the free, open-source program Blender (http://www.blender.org), a full-featured 3D modeling package. Once a three-dimensional model has been loaded into Blender, the user need only switch to the Edit Mode, triangulate the surface mesh of the desired object (Control-T after selection), and export the modified object as a collada DAE file. When a DAE file is provided, LipidWrapper will skip its own triangulation step entirely, relying instead of the surface triangulation specified by the file itself.

### Positioning Bilayer Models onto the Tessellated Triangles

**Figure 2:**
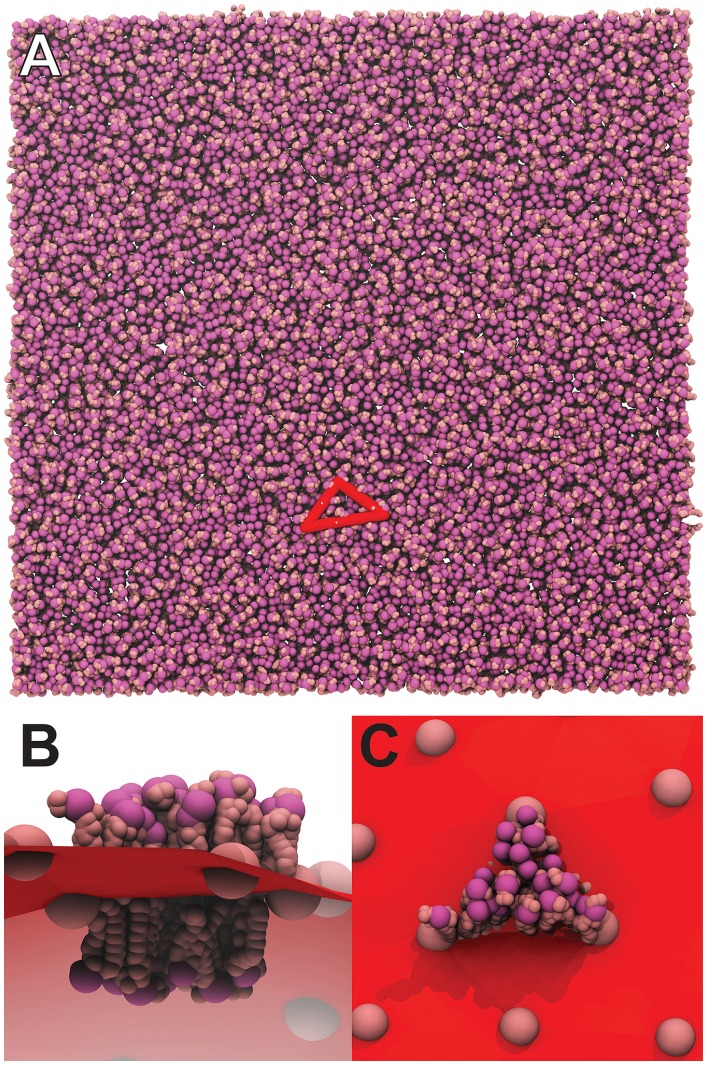
Positioning lipid triangles. A) Each of the tessellated triangles is projected onto a user-specified planar bilayer model. B and C) The lipid molecules that fall within each projected triangle are then translated back onto the three-dimensional surface.

With surface triangulation complete, LipidWrapper next uses components of PyMolecule [42], a python framework for loading and manipulating three-dimensional molecular models, to analyze a user-specified atomistic or coarse-grained PDB model of a planar lipid bilayer. The program assumes that the planar model is oriented parallel to the *x*–*y* plane, with the extracellular side facing the positive *z* direction (Figure 2A). The relevant PyMolecule definitions are fully integrated into LipidWrapper and so require no additional download or installation.

A sample planar bilayer model is included with the software, but different projects will likely require models with specific lipid compositions (e.g., for simulating *E. coli* membranes, nuclear membranes, etc.). A number of software packages are capable of generating planar lipid-bilayer models that can be used as LipidWrapper input. For example, CHARMM-GUI's Membrane Builder generates planar models with user-specified compositions [31].

Regardless of how the input model is obtained, the plane that bisects this bilayer model is next determined by averaging the *z* values of all lipid head groups, as judged by the position of the lipid phosphorus and cholesterol hydroxyl oxygen atoms (by default). Copies of the previously tessellated surface triangles are then randomly placed on this lipid-bisecting plane such that they are entirely contained within the region defined by the head groups (Figure 2A). The transformation matrices required to rotate and translate the triangle copies back onto their original 3D surface triangles are then calculated. These same matrices are applied to the lipid molecules that fall within each placed triangle, effectively positioning those lipids onto the three-dimensional surface (Figure 2B and 2C).

We note that the total surface area of the new bilayer model is dictated by the tessellated mesh, not by the dimensions of the initial planar bilayer model used as input. It is not that the initial bilayer is being geometrically distorted so that it surrounds the mesh; rather, an appropriately sized, randomly chosen section of the planar model is excised and superimposed on each mesh triangle. LipidWrapper-generated bilayer models typically have far more surface area than the planar models from which they are derived. To create this additional surface area, LipidWrapper may excise the same region of the initial planar model multiple times.

### Resolving Steric Clashes

**Figure 3:**
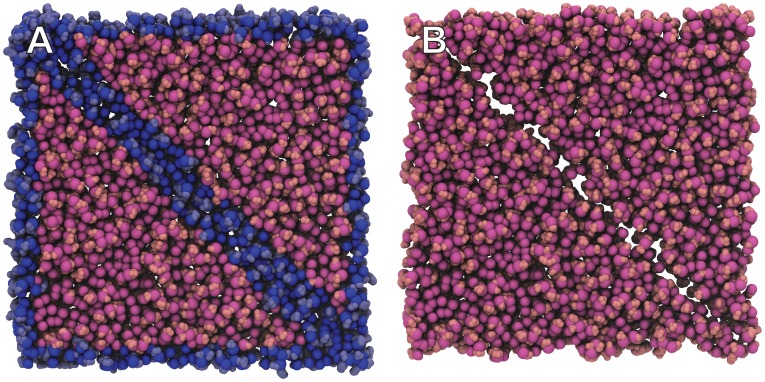
Removing lipid molecules that clash. The user can optionally instruct LipidWrapper to delete lipid molecules with steric clashes. A number of strategies are used to reduce the number of pairwise distance comparisons that must be performed. For example, A) only lipid molecules that are near the edges of their respective triangles (shown in blue) are evaluated for clashes. B) Clashing lipids are removed one by one, starting with the lipid that clashes with the greatest number of its neighbors, until all steric clashes have been resolved. Holes in the bilayer invariably result.

Next, the user can optionally instruct LipidWrapper to delete lipid molecules as needed to resolve steric clashes. As identifying steric clashes ultimately requires computationally expensive pairwise distance comparisons, the program goes to extraordinary measures to keep the number of distance calculations to a minimum. First, LipidWrapper assumes that lipids belonging to the same tessellated triangle do not clash, as they are all derived from a single bilayer model that was presumably carefully prepared. Second, as lipids belonging to distant tessellated triangles are unlikely to clash, LipidWrapper next seeks to identify proximate triangles that share corners (Figure 3) and/or have centers that come within a user-defined distance (50.0 Å by default). Third, as only lipids near triangle edges can clash, lipids are discarded if they have head groups that do not come within a user-specified distance of the triangle edge (25.0 Å by default) when projected onto their respective triangle planes (Figure 3A in purple/pink). Finally, as lipids with distant head groups are unlikely to clash, lipids from proximate triangles with head groups that are greater than a user-specified distance apart (50.0 Å by default) are likewise discarded.

Once the above pruning techniques have been completed, the remaining lipids, judged to be candidate clashers, are next considered. First, the program checks to see if the minimum coordinate of one lipid is so much larger than the maximum coordinate of its potential clash partner (in any dimension) so as to render a clash impossible. If not, all atoms (or coarse-grained “beads”) in the overlapping region of bounding boxes encompassing each lipid are identified. A pairwise distance comparison is then performed on these atoms, and if any two of them come within a user-specified distance (2.0 Å by default), the two lipids are judged to clash. Clashing lipids are removed one by one, starting with the lipid that clashes with the greatest number of its neighbors, until all steric clashes have been resolved (Figure 3B).

### Filling in the Resulting Bilayer Holes

**Figure 4:**
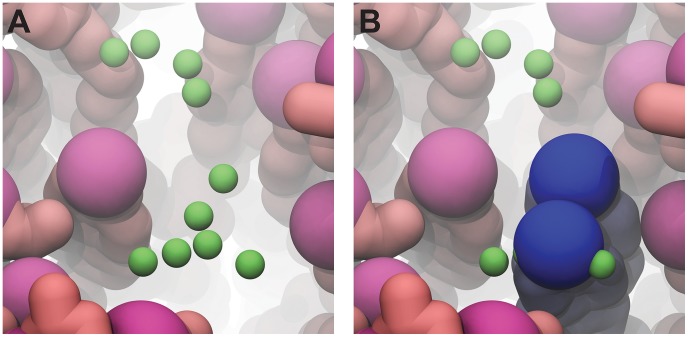
Filling in bilayer “holes.” A) Points (shown in green) are placed in bilayer holes at the level of the surrounding head groups. B) If steric clashes can be avoided, LipidWrapper then positions additional lipid molecules at these points (shown in blue).

This lipid-molecule deletion invariably produces “holes” in the bilayer. The user can optionally instruct LipidWrapper to fill these holes with additional lipid molecules. First, the region of each tessellated triangle near the triangle edges (where holes resulting from lipid deletions are likely to occur) is flooded with equidistant points. For each of these coplanar points, two new points are generated both above and below the triangle plane, positioned roughly at the level of the lipid head groups. After points too close to the surrounding lipids are deleted, the remaining points are effectively located in bilayer “holes” (Figure 4A, in green). LipidWrapper then iterates through each of these points a user-specified number of times (10 by default). A lipid molecule is placed at each point. If the new molecule does not clash with any of its neighbors, it is incorporated into the bilayer (Figure 4B, in blue).

### Subsequent Simulation

Although LipidWrapper is a program for building static bilayer models, we recognize that many will wish to use LipidWrapper-generated models in subsequent atomistic or coarse-grained molecular dynamics simulations. We ourselves have recently performed a brief simulation of a LipidWrapper-generated atomistic liposome model containing 4.3 million atoms and so can confirm that LipidWrapper bilayers are amenable to simulation. LipidWrapper preserves the residue and atom names used in the input planar-bilayer model. If the naming of the input model is consistent with a given force field (e.g., the CHARMM [43] or AMBER force fields [44]), the output model can be easily parameterized for that same force field using existing tools (e.g., the psfgen module of VMD [45], the LEaP module of AmberTools [46], etc.).

LipidWrapper's primary output is a single PDB file containing the entire large-scale model. However, for purposes of parameterization, it is often necessary to partition large models into segments. Fortunately, LipidWrapper also saves the individual planar patches associated with each tessellated triangle to separate PDB files, greatly facilitating parameterization.

## Results

### Using LipidWrapper to Gain Biological Insights into Influenza Virulence

To demonstrate how LipidWrapper can be used to provide novel biological insights, we modeled a patch of the influenza surface coat. Influenza is an enveloped virus with central ribonucleoproteins surrounded by a lipid membrane. Viral hemagglutinin (HA) and neuraminidase (NA) glycoproteins, consisting of enzymatic ectodomains anchored to the viral surface via filamentous stalks, both protrude roughly 15 nanometers from the membrane envelope [47]. Prior to infection, HA promotes cellular attachment by binding to sialic-acid residues on the carbohydrate side chains of cell-bound glycoproteins and glycolipids [48], [49]. After viral infection, replication, and budding, NA cleaves these sialylated oligosaccharide receptors, releasing the newly formed viral progeny so they can infect other cells [49], [50].

NA glycoproteins have varying heights as a result of deletions in the NA stalk. Studies have shown that virulence is mediated in part by this height, largely because short-stalk NA has reduced enzymatic activity against the cell-bound sialylated-oligosaccharide receptors, as demonstrated by impaired elution from erythrocytes [51], [52], [53]. The prevailing “limited access” theory proposes that shorter NA glycoproteins have reduced sialidase activity because they are buried beneath a towering canopy of surrounding HA glycoproteins; consequently, the cell-bound receptors cannot physically reach the NA enzymatic pockets [54], [55], [56]. As support for this theory, researchers have shown that NA activity is independent of stalk length if the small-molecule substrate MUNANA is used [57], [58]. In contrast, enzymatic activity against the bulky substrate fetuin is stalk-length dependent, apparently mimicking the activity of the endogenous receptor [53].

While this evidence is compelling, it is not conclusive. Neither MUNANA nor fetuin are particularly good mimics of the endogenous substrate. *In vivo*, this substrate is cell bound and so likely diffuses at a rate that differs substantially from that of an unbound small molecule like MUNANA. Differing rates of diffusion may have important impacts on overall binding kinetics/affinities. Additionally, fetuin (alpha-2-HS-glycoprotein) and the endogenous receptor are structurally distinct, and other studies have suggested that macromolecular bulk alone does not in fact prevent NA binding. For example, though their molecular masses are larger than that of fetuin, all available monoclonal antibodies known to target NA antigenic sites bind to NA regardless of stalk length [53], apparently unhindered by the steric influence of surrounding hemagglutinin glycoproteins.

**Figure 5:**
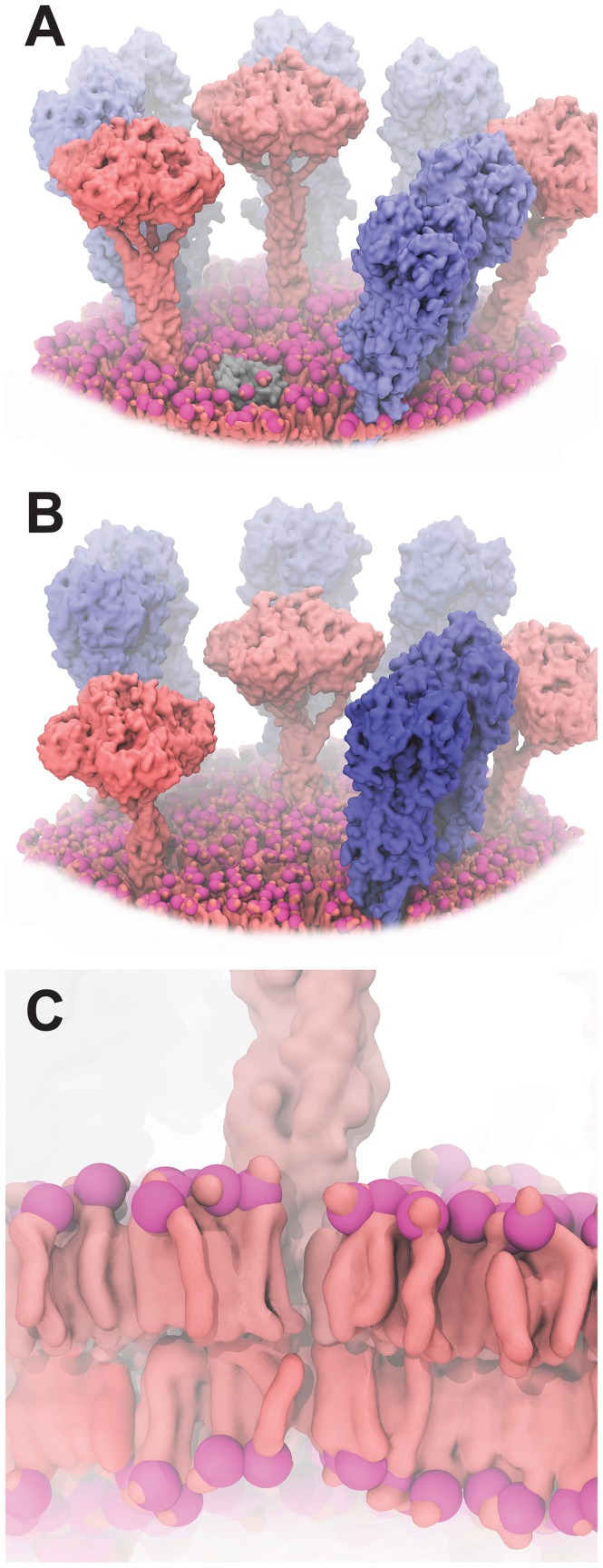
Models of influenza surface patches. NA and HA glycoproteins are shown in pink and blue, respectively. Large-scale curved bilayers were modeled using LipidWrapper. To improve clarity, some portions of the bilayers have been removed, and colors have been enhanced. A) A model including wild-type (long-stalk) NA. B) A model including a shorter NA, caused by a twenty-amino-acid deletion in the NA stalk. C) A close-up stylized view of the atomistic LipidWrapper-generated bilayer.

To examine the “limited access” theory *in silico*, we drew upon a 55 Å resolution cryo-electron tomography image [47] to generate two all-atom models of a virion-coat “patch” containing both HA and NA glycoproteins. Each patch contained wild-type (long-stalk, Figure 5A) or stalk-deletion (short-stalk, Figure 5B) NA models [59], respectively, extracted from molecular dynamics (MD) simulations that will be described in a forthcoming paper. In both cases, LipidWrapper was used to model the large-scale, curved lipid bilayer evident in the tomograms at full atomic-scale resolution. As the heights of the enzymatic glycoprotein heads are determined in large part by the position and orientation of the bilayer, proper bilayer modeling was critical in this case.

Visual inspection of these models calls into question the “limited access” theory (Figure 5). In part because of the curvature of the bilayer, the shorter NA (which in our models contains a substantial 20-amino-acid stalk deletion [59]) is in fact far more exposed to potential cell-bound receptors than one might otherwise assume. Other strains containing less substantial deletions that are nevertheless associated with enhanced virulence (i.e., the 2013 H7N9 strain with its five-amino-acid stalk deletion [60]) are likely even more exposed. The LipidWrapper-generated curved-bilayer component of these models was critical to this analysis, which ultimately suggests that the “limited-access” theory may not fully explain stalk-length-dependent NA activity *in vivo*.

### Bilayer-Model Resolution

Aside from high-resolution bilayer models like that of the influenza example above, in which each atom is explicitly represented, LipidWrapper can be used to produce coarse-grained models as well [61]. These models are comprised of “beads” that represent whole groups of atoms [62], [63] (i.e., a single bead might represent four adjacent lipid-tail carbon atoms, one or even several water molecules, a cholesterol ring, etc.). Both coarse-grained and atomic models have important advantages and disadvantages.

As coarse-grained models contain fewer individual particles, they are widely used to simulate large-scale systems, including systems with large membrane components [61], [64], [65], [66], [67]. Simulations of these simplified models can access timescales on the order of milliseconds [68] and so are emerging as useful tools for studying lipid phase transitions [69], protein interactions with lipid bilayers [70], lipid budding [64], etc. If the initial planar bilayer model used as LipidWrapper input is itself coarse grained, LipidWrapper generates coarse-grained models directly. Alternatively, atomistic models can be coarse grained after the fact using existing methods and tools (e.g., VMD's CGTools Plugin) [62], [71], [72], [73], [74].

While coarse graining certainly has its utility, many important biological questions can only be answered with atomistic models. For example, accurate electrostatics calculations require atomistic structures. When coupled with Brownian dynamics simulations, these calculations are useful for predicting small-molecule diffusion to a target receptor [75]. Similarly, studies of molecular recognition (e.g., docking) also often rely on atomic-resolution models (see, for example, refs. [76], [77], [78]).

Finally, when one wishes to study the detailed pharmacological impact of binding-pocket conformational sampling via MD simulations, the inaccuracies that result from coarse graining [79] can be problematic. Preliminary studies of hybrid atomistic/coarse-grained simulations, which allow certain portions of a large-scale system to be coarse grained while other portions remain atomistic, show some promise [80], [81], [82], [83]. In the future, it may become routine to coarse grain lipid-bilayer models while maintaining atomistic resolution at other sites of biological interest (e.g., the binding pockets of membrane-bound proteins).

### Comparison with Other Software Packages

To understand LipidWrapper's utility, consider the current state of the art. CHARMM-GUI's Membrane Builder, for example, is a useful resource for generating planar models [31] like those that LipidWrapper uses as input, but unlike LipidWrapper it cannot generate models of arbitrary geometry and, in our experience, is not well suited for building large-scale bilayer models. In contrast, assuming a sufficiently powerful machine is available, the size of LipidWrapper-generated bilayers is limited only by the constraints of the PDB file format itself, which allows at most 8 spaces for each X, Y, and Z atomic coordinate. Consequently, each PDB coordinate must be greater than −999.999 and less than 9999.999, permitting dimensions of at most 1.1 microns along each Cartesian axis. In preliminary benchmark testing, we successfully used LipidWrapper in conjunction with Visual Molecular Dynamics (VMD [45]) to create an atomistic bilayer model with a surface area of one square micron (320 million atoms, 4.2 gigabytes).

**Figure 6:**
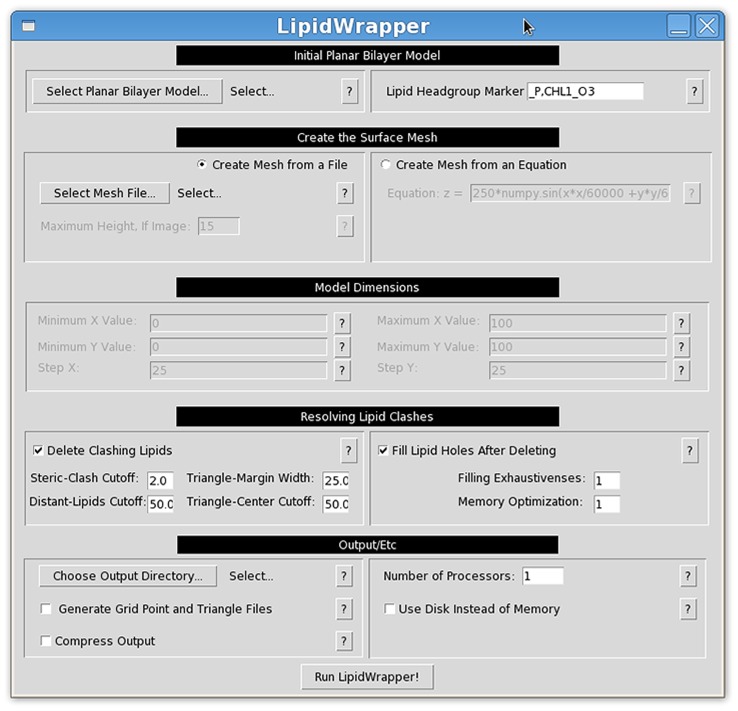
The LipidWrapper GUI. LipidWrapper includes an optional graphical user interface (GUI) to facilitate ease of use.

Packmol is another program worthy of mention. Like LipidWrapper, Packmol can generate bilayers with varied geometries. These geometries are determined by combinations of user-specified spheres, ellipses, cylinders, planes, and boxes [32] that are described programmatically in a script file. In contrast, LipidWrapper can generate bilayer geometries from many varied sources, both theoretical (e.g., from functions and 2D gray-scale images) and experimental (e.g., from PDB point files or DAE files). Knowledge of a program-specific scripting language is not required, and LipidWrapper provides a helpful GUI interface (Figure 6).

Finally, autoPack/cellPack [33], [34] perhaps most closely matches LipidWrapper's bilayer-generating abilities. autoPack is capable of packing any set of arbitrary shapes. The cellPack program specifically focuses on tightly packing biological shapes, including, thanks to recent additions to the code, the packing of lipid-shaped objects into membranes of varying geometries. If these lipid-shaped objects are derived from atomistic or coarse-grained lipid models, programs like UCSF Chimera [84] can be used to position the individual models within the cellPack-defined lipid-shape regions, thus producing a higher-resolution membrane model.

autoPack/cellPack is a useful model-building tool; however, LipidWrapper is better suited for generating bilayer models that are amenable to subsequent MD simulations. In our experience, bilayer models constructed lipid by lipid, such as those produced by cellPack, CHARMM-GUI, and Packmol, must often undergo extensive and sometimes tedious manual adjustments in order to resolve steric clashes and unrealistically tangled lipid tails before they can be used in simulations. In contrast, rather than assembling bilayers lipid by lipid, LipidWrapper generates large membrane models by tiling patches of smaller planar bilayers. Assuming these planar bilayers have been properly prepared, the only individual lipids that need be modified are those at the patch “seams,” where potential steric clashes are possible. The developers behind cellPack are aware of LipidWrapper's advantages and have even implemented our algorithm as a lipid-packing module within their own software.

## Availability and Future Directions

### Availability

LipidWrapper has been tested on all major operating systems with various versions of python, *numpy*, and *scipy* [35], [36], [37], [38], [39] (Table 1). Installation, execution, and usage instructions (Text S1), as well as specific examples of how large-scale bilayer models can be generated from functions, images, electron-tomography-derived point models, and blender collada DAE files (Text S2), are included in the Supplementary Materials. An up-to-date copy of the software can be downloaded from http://nbcr.ucsd.edu/lipidwrapper.

### Future Directions: Better Methods for Resolving Steric Clashes

Future directions will focus on developing techniques to better resolve occasional lipid-lipid steric clashes at the “seams” of adjacent tessellated triangles. As currently implemented, LipidWrapper optionally resolves steric clashes by deleting the offending molecules and filling the resulting holes with randomly selected lipids. While effective for many applications, this technique does not always perfectly resolve the occasional bilayer irregularities caused by molecule deletion.

MD simulations offer one potential solution. As LipidWrapper-generated bilayers are derived from pre-equilibrated planar bilayer models, they are already mostly equilibrated. Even brief simulations should resolve the occasional seam irregularities. For small-scale or coarse-grained models, brief equilibrating simulations are entirely tractable [85], [86], [87], [88]. If sufficient computer resources are available, simulations of more expansive atomistic models are also possible, though challenging [3].

To enable these equilibrating simulations, in the future we intend to develop helper scripts that generate the required MD input files. These scripts will first identify individual lipid molecules that are near triangle seams. If the user has previously instructed LipidWrapper to resolve steric clashes by deleing individual lipids, the resulting membrane holes can be “closed” by allowing seam-adjacent molecules to move freely during the simulation while fixing the positions of more distant lipid atoms. As most of the bilayer atoms will be fixed over the course of the simulation, the computational resources required to equilibrate will be significantly reduced.

Alternatively, if the user chooses to forgo LipidWrapper steric-clash resolution, lipid clashes could subsequently be eliminated by allowing seam-adjacent lipids to subtly expand over the course of an MD simulation. By exploiting software features commonly used to perform alchemical free-energy calculations like thermodynamic integration [89] and free energy perturbation [90], it may be possible to instruct an MD engine to slowly scale up the interactions between seam-adjacent and other (fixed) lipids from nonexistent to full over the course of a brief MD simulation [91]. This gradual scaling during simulation would resolve steric clashes without causing the dynamical instabilities and poor atomic geometries that might otherwise result from MD or minimization. As most of the LipidWrapper bilayer atoms would again be fixed, the computational cost would be reduced. Further efforts are required to explore the usefulness of these kinds of methods.

### Future Directions: Better Modeling the Membrane Asymmetries of Highly Curved Bilayers

Additionally, in the future we plan to develop methods to better model the membrane asymmetries of highly curved bilayers, where the surface area of the inner and outer leaflets are substantially different. *In vivo*, this difference may lead to asymmetries in head-group packing, lipid-tail disorder, and even leaflet composition [66]. Ideally, sufficiently long MD simulations might allow lipid molecules to equilibrate between the two monolayers via “flip-flop” [92]. This equilibration has in fact been observed in coarse-grained simulations of a small liposome, thanks in part to temporary artificial pores that were inserted into the bilayer to facilitate lipid translocation between membrane leaflets [66]. However, typically flip-flop occurs on time scales that are beyond those accessible with current MD techniques [92], [93], [94], necessitating alternative methods.

To address this challenge, we intend to create scripts for inserting and deleting specific lipids into the existing leaflets of LipidWrapper-generated membrane models, allowing us to fine-tune bilayer properties like lipid composition and area per head group. While these scripts will hardly serve as substitutes for full MD simulations, we believe they will help model builders create bilayer representations that are even truer to physiological reality than is currently possible.

### Conclusion

We are hopeful that LipidWrapper, which is open source and so can be easily expanded by others, will prove useful for computational biologists who wish to make more fluid connections across the molecular to subcellular scales of biological organization.

## Supporting Information

Figure S1 **Bilayers from functions.** LipidWrapper can generate bilayer models from mathematical functions. A) The mesh points (pink) and triangulation (red) derived from a user-specified function. B) The resulting bilayer model.
(TIF)

Figure S2 **Bilayers from two-dimensional images.** LipidWrapper can generate bilayer models from two-dimensional grayscale images. A) A smiley face was drawn by hand. A strong blur was applied to smooth the transitions between black and white. B) The mesh points (pink) and triangulation (red) derived from the user-specified image. C) The resulting bilayer model.
(TIF)

Figure S3 **Bilayers from PDB point files.** LipidWrapper can generate bilayer models from points specified in a PDB file. A) PDB-defined points representing the surface of an influenza virion, obtained by electron microscopy. The tessellation/triangulation is shown in red. B) The resulting bilayer model.
(TIF)

Figure S4 **Bilayers from collada DAE files.** LipidWrapper can generate bilayer models from collada DAE files exported from Blender. A) The mesh points and tessellation/triangulation, as visualized in Blender. B) The resulting LipidWrapper bilayer model.
(TIF)

Text S1 **Installation, documentation, and usage instructions.**
(DOCX)

Text S2 **Examples of how large-scale bilayer models can be generated from functions, images, electron-microscopy-derived point models, and Blender collada DAE files.**
(DOCX)

Software S1 **The LipidWrapper program, with sample data included.**
(ZIP)
