# Supplementary figures and images for: LipidWrapper: An Algorithm for Generating Large-Scale Membrane Models of Arbitrary Geometry
Source: PLoS Comput Biol. 2014 Jul 17;10(7):e1003720. doi: 10.1371/journal.pcbi.1003720 (PMC4102414; doi:10.1371/journal.pcbi.1003720)

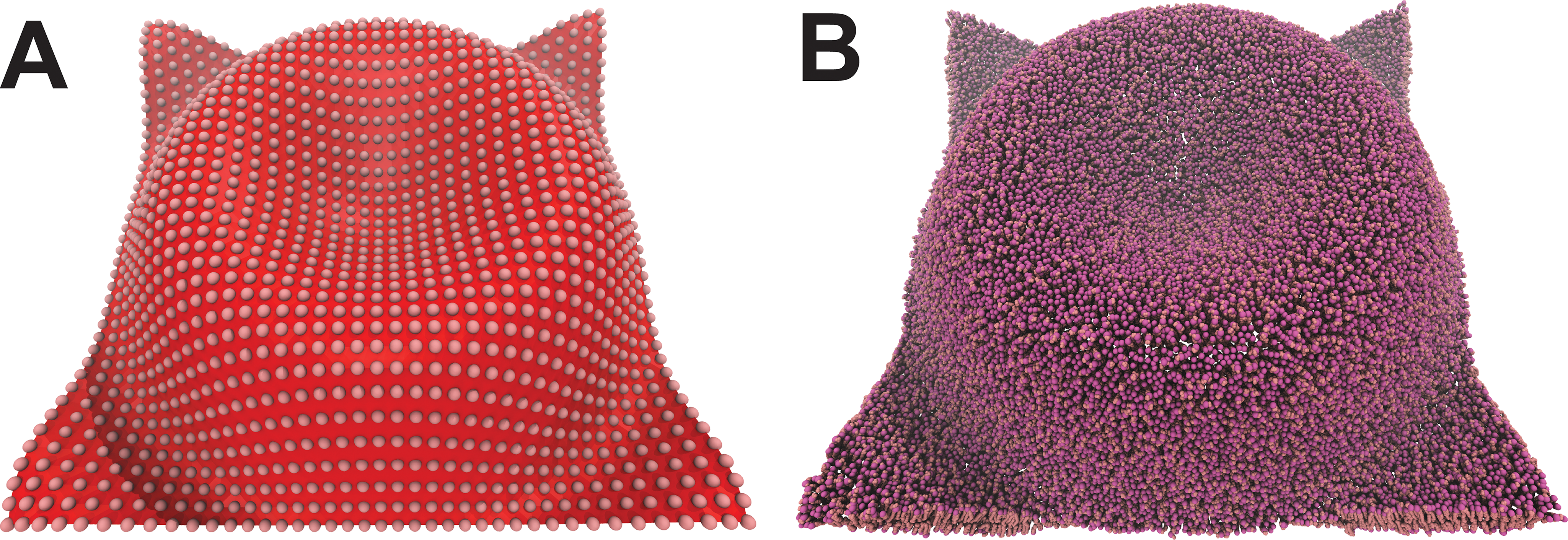

Supplement: Figure S1 — Bilayers from functions. LipidWrapper can generate bilayer models from mathematical functions. A) The mesh points (pink) and triangulation (red) derived from a user-specified function. B) The resulting bilayer model. (TIF) [file pcbi.1003720.s001.tif]

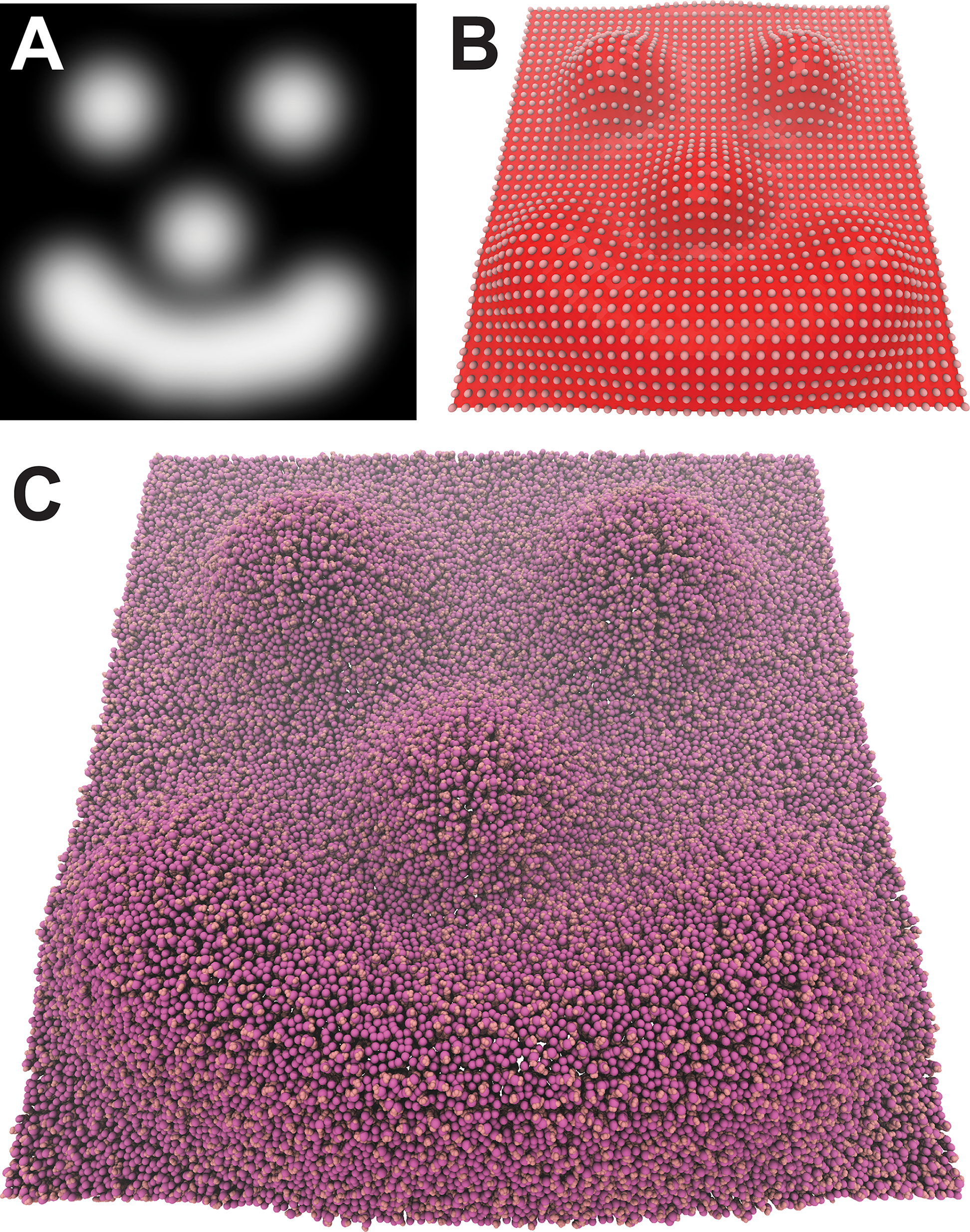

Supplement: Figure S2 — Bilayers from two-dimensional images. LipidWrapper can generate bilayer models from two-dimensional grayscale images. A) A smiley face was drawn by hand. A strong blur was applied to smooth the transitions between black and white. B) The mesh points (pink) and triangulation (red) derived from the user-specified image. C) The resulting bilayer model. (TIF) [file pcbi.1003720.s002.tif]

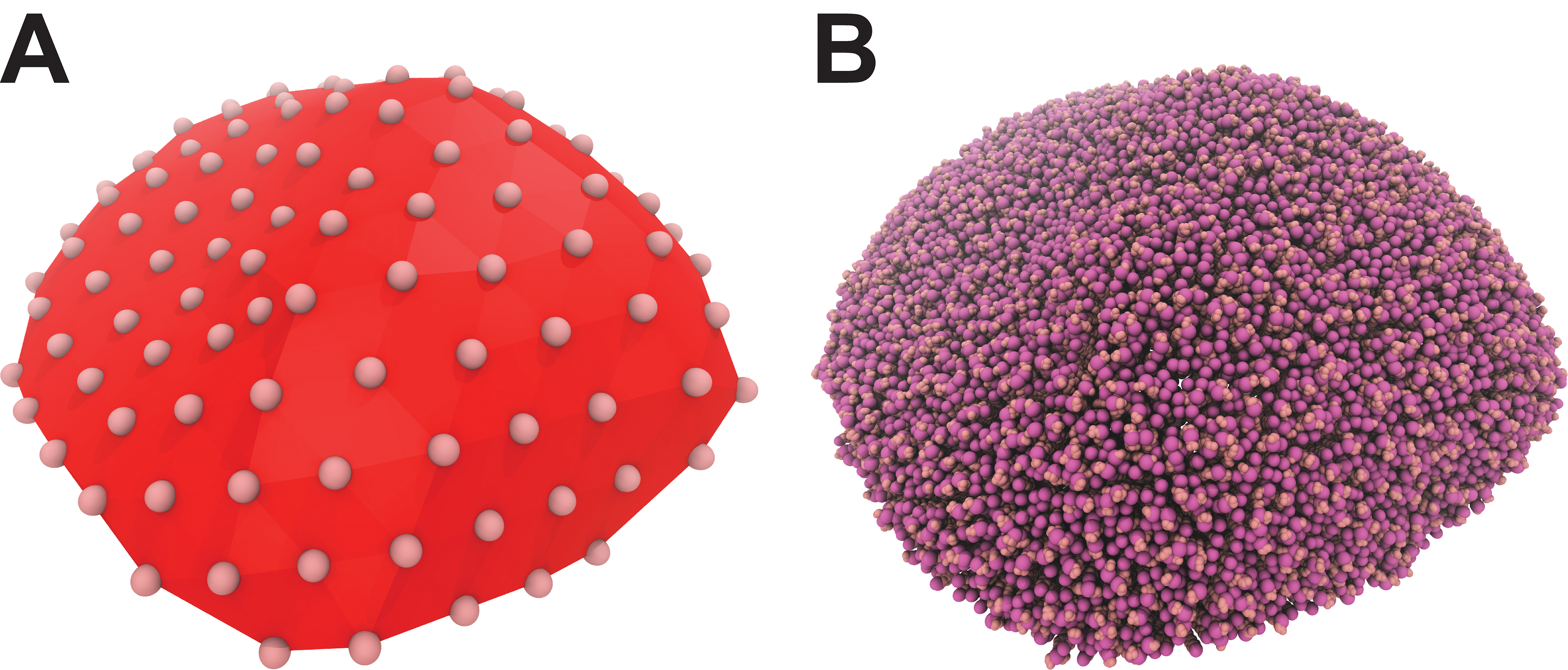

Supplement: Figure S3 — Bilayers from PDB point files. LipidWrapper can generate bilayer models from points specified in a PDB file. A) PDB-defined points representing the surface of an influenza virion, obtained by electron microscopy. The tessellation/triangulation is shown in red. B) The resulting bilayer model. (TIF) [file pcbi.1003720.s003.tif]

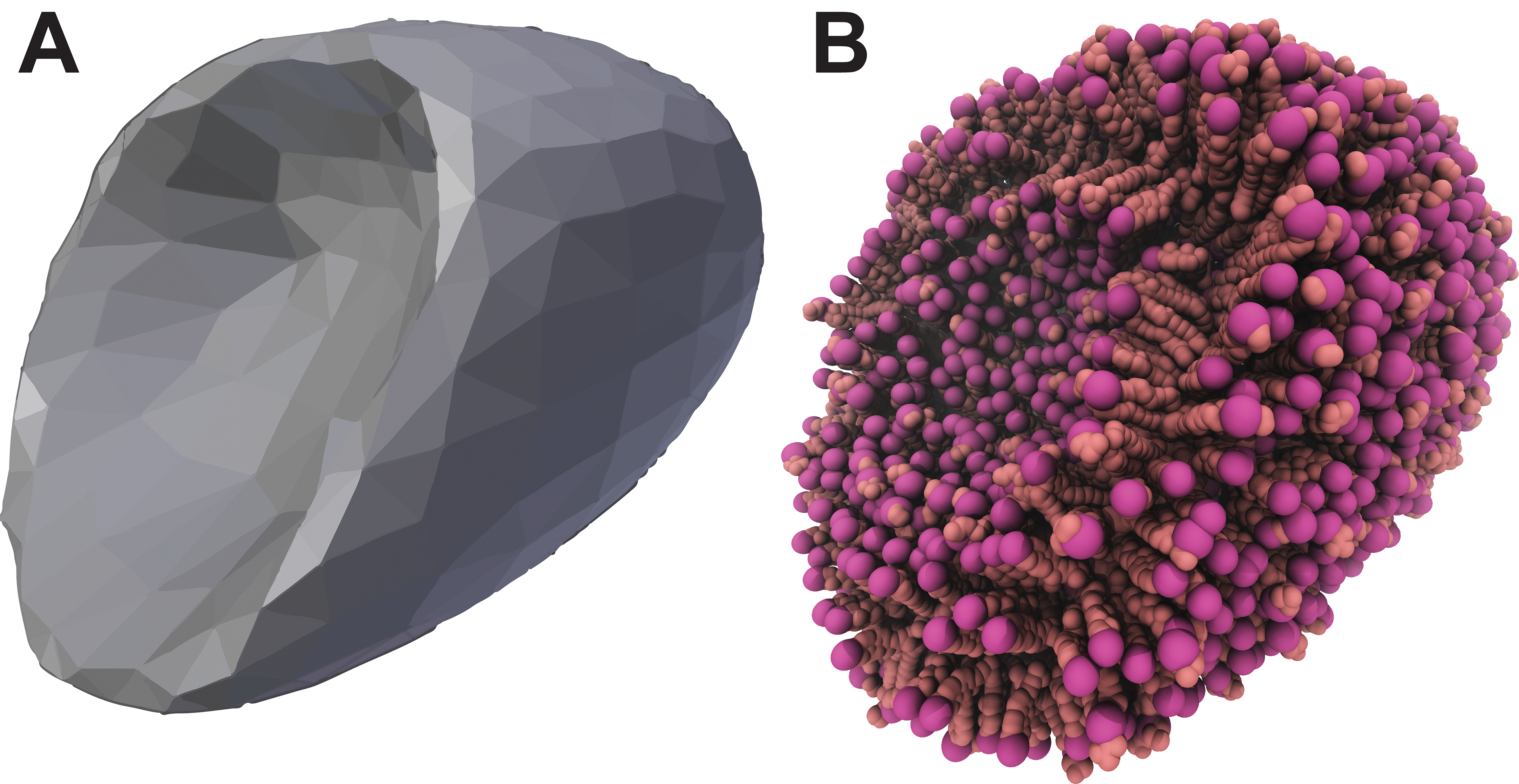

Supplement: Figure S4 — Bilayers from collada DAE files. LipidWrapper can generate bilayer models from collada DAE files exported from Blender. A) The mesh points and tessellation/triangulation, as visualized in Blender. B) The resulting LipidWrapper bilayer model. (TIF) [file pcbi.1003720.s004.tif]

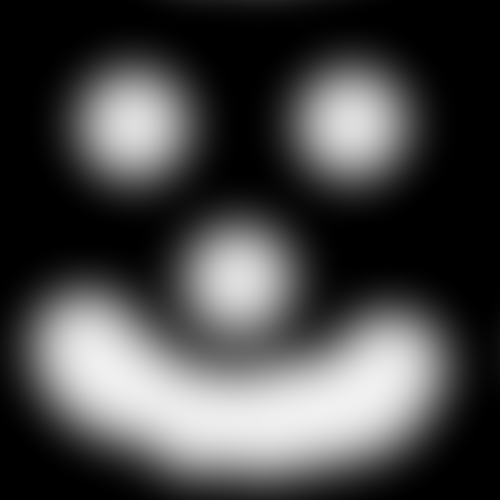

Supplement: Software S1 — The LipidWrapper program, with sample data included. (ZIP) [file pcbi.1003720.s007.zip › lipidwrapper/examples/files/face.png]

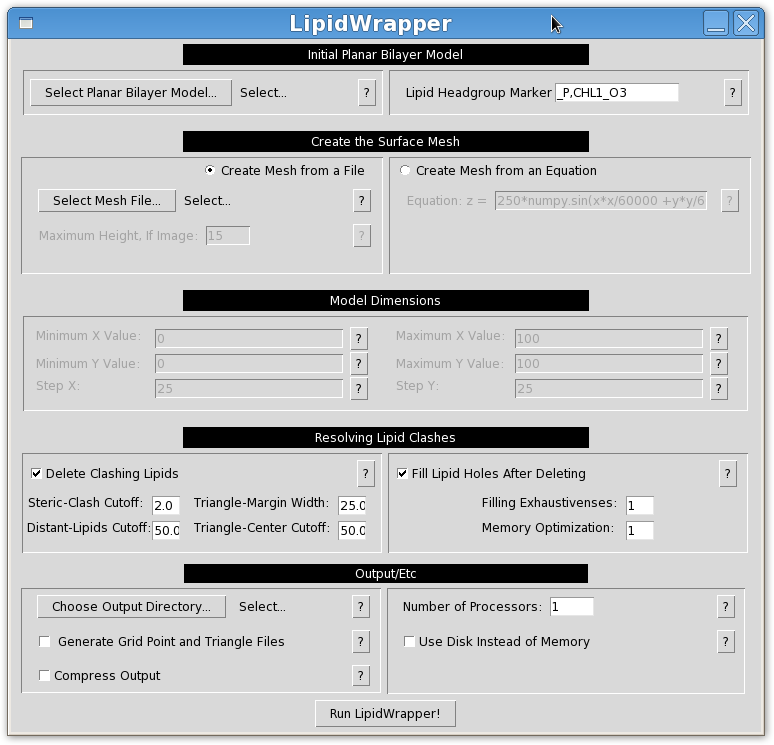

Supplement: Software S1 — The LipidWrapper program, with sample data included. (ZIP) [file pcbi.1003720.s007.zip › lipidwrapper/Screenshot.png]
