# Supplementary material for: LipidWrapper: An Algorithm for Generating Large-Scale Membrane Models of Arbitrary Geometry
Source: PLoS Comput Biol. 2014 Jul 17;10(7):e1003720. doi: 10.1371/journal.pcbi.1003720 (PMC4102414; doi:10.1371/journal.pcbi.1003720)
Supplement: Text S1 — Installation, documentation, and usage instructions. (DOCX) [file pcbi.1003720.s005.docx]

**How to Install and Run the Software**

As a python script, LipidWrapper should run on any operating system that has python, *numpy*, and *scipy* installed, without requiring the installation of additional software. If the user optionally wishes to generate lipid-bilayer models from PNG images, the Python Imaging Library must also be installed. LipidWrapper has been explicitly tested on Scientific Linux 6.2, OS X, Windows XP, and Windows 7.

The program download includes an “examples” directory that demonstrate how to use the software. Examples are provided showing how to generate lipid-bilayer models wrapped around equations, PDB point files, DAE models, and image-defined surfaces. Test PDB, DAE, and PNG files are included.

**Documentation**

The documentation is included in the README text file, distributed with the software. For convenience, a portion of the README file is reproduced here.

Command-Line Parameters

=======================

Methods for creating a surface mesh

===================================

--surface_equation: Generate a surface mesh from a python-formatted

equation defining z, given x and y. The --min_x, --max_x,

--min_y, and --max_y parameters are used to specify the region

over which the function should be evaluated. The --step_x and

--step_y parameters define the x-y distance between adjacent

points. Python functions from the math, numpy, and scipy modules

can be used. Example: --surface_equation "z =

250*numpy.sin(x*x/60000 +y*y/60000)"

--surface_filename: If this parameter specifies a file with the PDB

extension, a surface mesh is generated from the coordinates of

the PDB atoms. Example: --surface_filename mymesh.pdb

--surface_filename: If this parameter specifies a file that does not

have the PDB extension, the file is assumed to be a gray-scale

image, where black represents regions that are topologically

low, and white represents regions that are topologically high.

The --min_x, --max_x, --min_y, and --max_y parameters are used

to specify the region where the mesh should be generated. The

--step_x and --step_y parameters define the x-y distance between

adjacent points. The --max_height parameter determines the

height of the bilayer model at those locations where the image

is white; black regions are assigned a height of 0. This feature

is only available if the python PIL module has been installed on

your system. Example: --surface_filename mymesh.png

The initial lipid model

=======================

--lipid_pdb_filename: This parameter specifies a PDB file containing

an all-atom model of a planar lipid bilayer. LipidWrapper will

wrap this lipid around the user-generated mesh. Example:

--lipid_pdb_filename lipid.pdb

--lipid_headgroup_marker: A unique atom representing the headgroup of

each lipid residue must be specified. The

--lipid_headgroup_marker accepts a comma-separated lists of atom

specifications (RESNAME_ATOMNAME). If either RESNAME or ATOMNAME

is omitted, any value will be accepted. By default, LipidWrapper

identifies lipid headgroups by looking for any atom named "P"

(_P) or any atom named "O3" belonging to a cholesterol molecule

(CHL1_O3). Example: --lipid_headgroup_marker "_P,CHL1_O3"

Methods for resolving lipid clashes

===================================

--delete_clashing_lipids: It's common for lipids to sterically clash

at the interface of two adjacent surface-mesh tessellated

triangles. If this parameter is set to TRUE, any clashing lipids

are deleted. Example: --delete_clashing_lipids TRUE

--clash_cutoff: If you do choose to delete clashing lipids, this

parameter determines how close two atoms must be (in Angstroms)

to constitute a steric clash. Example: --clash_cutoff 2.0

--fill_holes: Deleting lipids often leaves holes in the membrane. If

this parameter is set to TRUE, LipidWrapper tries to fill the

hole. Example: --fill_holes TRUE

--fill_hole_exhaustiveness: Essentially, how long LipidWrapper should

try to fill the holes. Example: --fill_hole_exhaustiveness 10

--clashing_potential_margin: Lipid clashes occur at the edges of

adjacent tessellated triangles. If these triangles are very

large, it's faster to only check for clashes and holes near the

triangle edges. This variable specifies how far from the edges,

in Angstroms, that LipidWrapper should look for clashes and

holes. Example: --clashing_potential_margin 25.0

--very_distant_lipids_cutoff: LipidWrapper determines if two lipids

clash by comparing the distance between every atom in the first

lipid with every atom in the second lipid. This can be

computationally expensive. However, sometimes two lipids are so

distant from each other, that it's obvious there are no clashes,

making the pair-wise comparison unnecessary. Before performing

this expensive pair-wise comparison, LipidWrapper calculates the

distance between one atom of each lipid. If this distance is

greater than this user-specified cutoff, the program will simply

assume there are no clashes. WARNING: Remember to consider the

width of your lipid bilayer when choosing this value. Adjacent

lipids on opposite sides of the bilayer can seem distant when

considering the distance between their headgroups, for example.

Example: --very_distant_lipids_cutoff 50.0

--triangle_center_proximity_cutoff_distance: Lipid steric

clashes/holes typically occur between lipids that belong to

adjacent tessellated triangles. However, if tessellated

triangles are small enough, clashes are possible between lipids

that belong to non-adjacent triangles as well. Consequently, in

addition to checking for adjacency, LipidWrapper also checks the

distance between the triangle centers, using this user-specified

value as a cutoff. Example:

--triangle_center_proximity_cutoff_distance 50.0

--memory_optimization_factor: When the tessellated triangles are very

large and consequently contain many individual lipids, the

extensive pairwise distance comparisons required can result in

memory errors. This parameter tells lipid Wrapper to divide the

list of atoms being compared into smaller chunks. The pairwise

distance comparison is performed piecewise on each chunk-chunk

pair and so uses less memory, albeit at the expensive of speed.

Only increase the value of this parameter if you run into memory

errors. Example: --memory_optimization_factor 1

Additional options

==================

--number_of_processors: Using multiple processors can significantly

increase the speed of the LipidWrapper algorithm. Example:

--number_of_processors 8

--show_grid_points: Aside from producing PDB coordinates for lipid

atoms, additional coordinates will be appended to the bottom of

the output containing "atoms" named "X" that specify the

location of the surface mesh points. Example: --show_grid_points

TRUE

--create_triangle_tcl_file: A separate file named "triangles.tcl" will

be generated containing a tcl script that can be run in VMD to

visualize the mesh surface. Example: --create_triangle_tcl_file

TRUE

--output_directory: If an output directory is specified, all

LipidWrapper output files, as well as additional files

representing the intermediate steps required to build the final

bilayer, will be saved in that directory. Example:

--output_directory ./my_output/

--use_disk_instead_of_memory: For very large systems, storing the

growing model in memory can be problematic. If this parameter is

set to TRUE, the growing model will be stored on the hard disk

instead. However, expect longer execution times if this

parameter is set to TRUE. Example: --use_disk_instead_of_memory

TRUE

--compress_output: Depending on the user options selected,

LipidWrapper output can require a lot of disk space. If this

parameter is set to TRUE, the output will be automatically

compressed using the gzip algorithm (Lempel-Ziv coding LZ77).

The files can be uncompressed with the UNIX gunzip utility, or

similar Windows-based packages. Example: --compress_output TRUE

Example

=======

python lipidwrapper.py --surface_equation "z = 250*numpy.sin(x*x/60000

+y*y/60000) * (-numpy.sqrt(x*x+y*y)/(560 * numpy.sqrt(2)) + 1)"

--min_x 500 --max_x 1000 --min_y 500 --max_y 1000 --step_x 25

--step_y 25 --lipid_pdb_filename lipid.pdb

--lipid_headgroup_marker "_P,CHL1_O3" --delete_clashing_lipids

TRUE --clash_cutoff 1.0 --fill_holes TRUE

--fill_hole_exhaustiveness 10 > lipid_model.pdb
