# Supplementary material for: LipidWrapper: An Algorithm for Generating Large-Scale Membrane Models of Arbitrary Geometry
Source: PLoS Comput Biol. 2014 Jul 17;10(7):e1003720. doi: 10.1371/journal.pcbi.1003720 (PMC4102414; doi:10.1371/journal.pcbi.1003720)
Supplement: Text S2 — Examples of how large-scale bilayer models can be generated from functions, images, electron-microscopy-derived point models, and Blender collada DAE files. (DOCX) [file pcbi.1003720.s006.docx]

**Examples of LipidWrapper Usage**

*Fitting a Bilayer to an Equation*

We first generated a mesh of surface points from the equation:


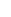


for *x* and *y* ranging from -500 to 500 Å. Mesh points were spaced 25 Å apart in both the *x* and *y* directions (Figure S1A). Any python equation can be used to define *z*. The actual code, provided as a command-line parameter, was "z = 250 * numpy.sin(x * x/60000 +y * y/60000) * (-numpy.sqrt(x * x+y * y)/(560 * numpy.sqrt(2)) + 1)". We note that LipidWrapper imports the *math*, *numpy*, and *scipy* modules; python functions from any of these modules can be used to define *z*.

An atomistic lipid-bilayer model containing cholesterol, phosphatidylserine, phosphatidylcholine, phosphatidylethanolamine, and phosphatidylglycerol molecules was tiled across the surface defined by these points, as described in the Methods section, generating a bilayer model that ultimately contained 3,085,485 atoms (Figure S1B).

*Fitting a Bilayer to an Image*

We next generated a surface mesh from an image. The general shape of the bilayer was drawn in the GNU Image Manipulation Program (GIMP) [1], version 2.6.9. A square canvas with sides 500 pixels long was initially colored black, representing the "low" region of the lipid topology. A smiley face was then drawn over this black background in white, representing "high" regions of the lipid. In order to remove discontinuities, a strong blur was applied to the white portion of the image, which effectively smoothed the transitions between black and white. The resulting image was saved as a PNG file (Figure S2A).

As the Python Imaging Library (PIL) [2] had been installed on our system, we were able to use LipidWrapper's image-loading feature to generate a mesh from this PNG file. LipidWrapper generated mesh points with *x* and *y* values ranging from -500 to 500, spaced 25 Å apart. The maximum height of the mesh, 150 Å, corresponded to a color of pure white in the gray-scale PNG. Regions of pure black were assigned heights of 0 Å (Figure S2B).

The same planar lipid-bilayer model used previously was again employed. The resulting smiley-face-shaped bilayer, which ultimately contained 2,981,257 atoms, is shown in Figure S2C.

*Fitting a Bilayer to a Mesh Defined by PDB Points*

To demonstrate how LipidWrapper can be used to model a real-world bilayer, we obtained a PDB file from an experimental collaborator containing points that span the surface of an influenza virion, as determined by electron microscopy (Figure S3A) [3]. Influenza is an enveloped virus, meaning its outermost layer, represented by these points, is a lipid bilayer. We selected a limited region of the PDB mesh model and, using the same planar lipid model described above, again generated a larger, curved bilayer model that ultimately contained 737,637 atoms (Figure S3B).

*Fitting a Bilayer to a Triangulated Mesh Generated in Blender*

To demonstrate how the 3D modeling program Blender can be used together with LipidWrapper, we first created a spherical "meat ball" in the Blender GUI. This meat ball was expanded to an appropriate size and deformed, producing a concave indentation on the enclosed surface (Figure S4A). The surface of the mesh was then triangulated in Blender, and the object was exported as a collada DAE file [4]. The collada format was chosen for LipidWrapper input because it is text based, human readable, and XML formatted, and so can be easily loaded and manipulated in Python. LipidWrapper was run using the same DAE file as input. As this file includes both mesh-point locations and triangulations, LipidWrapper skipped its own triangulation step.

The resulting curved bilayer contained 283,709 atoms and is shown in Figure S4B. Lipid-positioning artifacts present at triangle-triangle interfaces with sharp edges (i.e., regions where the tessellated triangles do not approximate a differentiable surface) have been retained to demonstrate the importance of providing LipidWrapper with smooth, physiologically relevant bilayer surfaces. The program positions lipid headgroups above and below each triangle plane. If adjacent triangles do not have roughly equivalent normal vectors, breaks in the bilayer will result. The same may true of unnaturally curved bilayers *in vivo*; natural bilayers tend to be smooth due to their relatively large surface tensions.

**References**

1. GIMP G (2004) Image Manipulation Program. Freely distributed Image Processing Software <http://www> gimp org.

2. Lundh F, Ellis M (2012) Python Imaging Library (PIL). Secret Labs AB, <http://www>. pythonware. com/products/pil.

3. Fontana J, Cardone G, Heymann JB, Winkler DC, Steven AC (2012) Structural Changes in Influenza Virus at Low pH Characterized by Cryo-Electron Tomography. Journal of Virology 86: 2919-2929.

4. Barnes M, Finch E (2008) COLLADA-3D Asset Exchange Schema, Release 1.5.0. Khronos Group.
